# Malignant Transformation in Glioma Steered by an Angiogenic Switch: Defining a Role for Bone Marrow-Derived Cells

**DOI:** 10.7759/cureus.471

**Published:** 2016-01-27

**Authors:** Raymond Xu, David Pisapia, Jeffrey P Greenfield

**Affiliations:** 1 Neurological Surgery, Weill Cornell Medical College; 2 Pathology, Weill Cornell Medical College; 3 New York Presbyterian Hospital

**Keywords:** glioma, low-grade glioma, gbm, recurrent glioblastoma, high-grade glioma, malignant transformation, angiogenesis, neovascularization, angiogenic switch, bone marrow-derived cells

## Abstract

Low-grade gliomas, such as pilocytic astrocytoma and subependymoma, are often characterized as benign tumors due to their relative circumscription radiologically and typically non-aggressive biologic behavior. In contrast, low-grades that are by their nature diffusely infiltrative, such as diffuse astrocytomas and oligodendrogliomas, have the potential to transform into malignant high-grade counterparts and, given sufficient time, invariably do so. These high-grade gliomas carry very poor prognoses and are largely incurable, warranting a closer look at what causes this adverse transition. A key characteristic that distinguishes low- and high-grade gliomas is neovascularization: it is absent in low-grade gliomas, but prolific in high-grade gliomas, providing the tumor with ample blood supply for exponential growth. It has been well described in the literature that bone marrow-derived cells (BMDCs) may contribute to the angiogenic switch that is responsible for malignant transformation of low-grade gliomas. In this review, we will summarize the current literature on BMDCs and their known contribution to angiogenesis-associated tumor growth in gliomas.

## Introduction and background

### Current standard of treatment for low-grade gliomas

Gliomas exist as a spectrum of low- and high-grade states and are classified as Grade I, II, III, or IV, according to the 2007 World Health Organization (WHO). Low-grade infiltrating gliomas are currently assigned a WHO grade of II and are characterized by relatively low cellularity, mild to moderate nuclear pleomorphism, a low proliferative index, and the absence of microvascular proliferation that often correlates with non-enhancing radiological imaging. The median survival projection and 10-year survival rate for low-grade diffuse fibrillary astrocytomas are 4.7 years and 17%, respectively, while oligodendrogliomas confer a slightly better prognosis with 7.1 years and 33% survival [1]. The management of low-grade gliomas has been a challenging topic in clinical neuro-oncology and is contingent on numerous factors, including clinical presentation, imaging characteristics, clinician opinion and experience, and patient goals [2]. Although gross total resection is the ideal treatment for low-grade tumors amenable to surgery, this option is not always attainable and is based upon the intracranial location of the mass and possible morbidity associated with a resection. Gross total resection demonstrates the greatest five-year overall survival rate at 63% compared to a 27% rate with subtotal resection [3]. Adjuvant therapies, such as radiation therapy and chemotherapy, are approached with similar prudence, given the toxicity of such treatments. While they may remain indolent for years, Grade II gliomas possess the potential to progress to their Grade III and IV malignant high-grade counterparts, which are aggressive and invasive. The most ominous of these tumors, glioblastoma (GBM), carries a dismal prognosis of an 18-month median survival even with the most aggressive treatments due to inevitable recurrence. These high-grade astrocytic tumors are highly pleomorphic, present a high degree of proliferation, and are characterized by contrast enhancement on MRI. They are also characterized by necrosis, microvascular proliferation, and hemorrhage, and it is postulated that neovascularization in high-grade gliomas may provide the tumor with the blood supply necessary for further growth and proliferation. It has been hypothesized that low-grade tumors encounter a phenomenon known as the angiogenic switch that permits rapid progression and transformation toward malignant high-grade glioma [4].

## Review

### Low-grade to high-grade transition characterized by neovascularization

The link between tumor growth and invasiveness, and angiogenesis has been well-documented [5]. It is logical that the ability of solid tumors to recruit neovasculature would be a rate-limiting step in tumor progression because, as with all cell types, growth depends upon nutrients provided by circulating blood. The spectrum of low- to high-grade gliomas may be a perfect proof of this concept, as neovascularization is absent in clinically indolent low-grade tumors, but represents a prominent feature of malignant high-grade tumors, including high-grade astrocytoma, oligodendrogliomas, and ependymomas. It has been theorized that the relative lack of vascularization in low-grade tumors confines it to a linear growth model [6], whereas the hypervascularization present in GBM allows for exponential growth and subsequent rapid clinical decline [7]. While neovascularization is a normal process that occurs by way of ischemic stimuli, tumors can hijack the machinery to promote growth and invasiveness, activating the angiogenic switch [8]. In this mechanism, signals, such as vascular endothelial growth factor (VEGF), platelet-derived growth factor (PDGF), and hypoxia-inducible factor 1-alpha (HIF-1α), are released from glioma stem cells in low oxygen states [9], causing proteases to detach pericytes from existing vessels and forming a weak extracellular matrix around the vessel wall that promotes endothelial cell remodeling [10]. This ultimately promotes not only the branching of existing vessels (angiogenesis) but also the formation of *de novo* vessels (vasculogenesis). There is evidence that high-grade tumors have transcriptional alterations that correlate with neovascularization, and the upregulation of these genes may also play a role in activating the angiogenic switch. Godard, et al. observed upregulation in several angiogenesis-associated genes, such as VEGF, fibroblast growth factor (FGF), and epidermal growth factor (EGF), in human GBM samples as compared to low-grade astrocytoma [11]. It has also been demonstrated that these factors mobilize a set of BMDCs to the site of neovascularization to further augment the angiogenic effect [12]. Given the limited success of current anti-angiogenic therapies in high-grade gliomas [13], BMDCs offer an additional target to prevent neovascularization and subsequently prevent the malignant transformation of low-grade gliomas. In order to elucidate potential areas of study, we must first characterize what we already know about BMDCs.

### Implication of BMDC contribution in tumors

**Figure 1:**
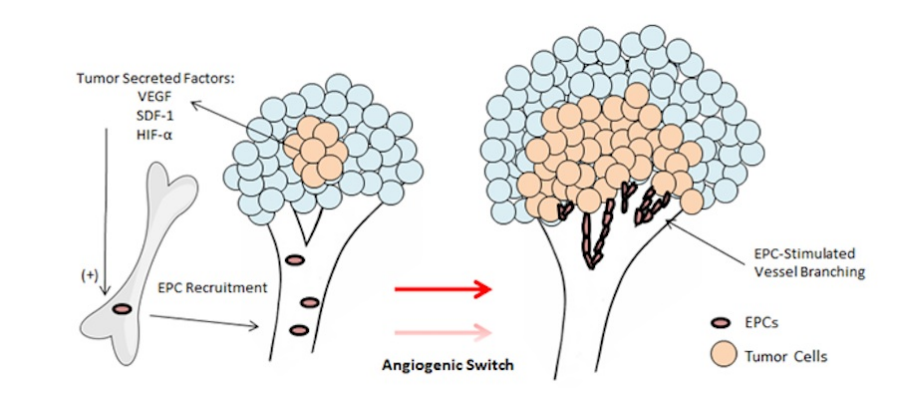
Tumor-derived factors recruit endothelial progenitor cells to tumor site, activating the angiogenic switch necessary for malignant transformation Adapted from Rafii & Lyden [4].

BMDCs are a heterogeneous population of cells comprising endothelial (EPCs) and hematopoietic precursor cells (HPCs), mesenchymal stem cells (MSCs), myeloid-derived suppressor cells (MDSCs), Tie-2 expressing monocytes (TEMs), and tumor-associated macrophages (TAMs). While the exact role of each subset has not been precisely defined in the context of neovascularization and tumor invasion, it has been shown that BMDCs do in fact help create a niche capable of sustaining tumor growth by breaking down normal structures to promote neovascularization and tumor proliferation. This has been supported by a well-described contribution of BMDCs in metastatic disease [14] where bone marrow precursors have been found mobilized and directed to metastatic sites prior to tumor formation, perhaps acting as a primer for tumor invasion. In the brain, mobilization of bone marrow precursors to the tumor has also been demonstrated to promote vasculogenesis in gliomas through a hypoxia-induced mechanism [15]. Again, we revisit the idea that neovascularization is triggered by hypoxic stimuli, occurring most relevantly in the hypoxic tumor environment. Du, et al. showed that release of VEGF and HIF-1α by tumor cells, in combination with stromal cell-derived factor 1 (SDF-1) and C-X-C chemokine receptor type 4 (CXCR-4) receptor activation, spurred mobilization of BMDCs to the invasive tumor front. Specifically, the recruitment of EPCs and myeloid cells to the tumor site induced a pro-angiogenic state [16]. This led to speculation that radiation may play a role in tumor recurrence by way of hypoxia-induced vasculogenesis [17]. Given the significance of neovascularization in malignant transformation, and the reported contribution of BMDCs in angiogenesis, these findings offer further support for the theory that BMDCs play a role in the angiogenic switch thought to cause malignant transformation (Figure 1).

### Clinical implications of BMDC involvement in transformation

Molecular profiling of various BMDC subsets has helped to identify and characterize the populations of cells involved in clinical incidences of malignant progression. Quantifying these populations holds substantial therapeutic implications. EPCs, which have been phenotypically described as CD133^+ ^and VEGFR2^+^ cells, have been found in the peripheral blood of malignant astrocytoma patients [18]. Interestingly, the presence of this population of precursor cells was significantly increased in brain tumor patients compared to normal controls, and also correlated with tumor grade and predicted survival [19-20]. This finding was congruous with previous animal studies using a PDGF-driven murine model of GBM that closely models a low-grade to high-grade transition. In those studies, specific populations of BMDCs were found in the peripheral blood of mice with high-grade tumors but not in mice bearing low-grade tumors [20]. Clinically, a CD11b^+^ MDSC population has demonstrated the capability to distinguish between low and high-grade tumors (unpublished data). Immunofluorescent staining indicated both highly prevalent CD11b^+^ cells in human GBM and aberrant (CD144^+^-labeled) vessel formation that was significantly reduced in comparable human Grade II astrocytomas (unpublished data). As previously stated, myeloid cells have been known to contribute to a pro-angiogenic environment, however, the CD11b^+^ subset also has been widely associated with inflammation and anti-tumor immunosuppression mechanisms involved with malignant gliomas, notably GBM [21], implicating it as a potentially significant factor in malignant progression. Further characterizing BMDC subsets in low-grade glioma patients may provide the necessary biomarkers to measure the potential for tumor angiogenicity and malignancy, ultimately increasing our ability to predict the likelihood of malignant transformation.

## Conclusions

It is apparent that the angiogenic switch plays a crucial role in the malignant transition of lower grade diffusely infiltrating gliomas to their high-grade counterparts, in part by providing the circulating nutrients required for exponential growth. Anti-angiogenic therapies more directly targeting vascular elements themselves have shown limited clinical efficacy; thus, our attention has shifted to a population of bone marrow-derived precursor cells that have been demonstrated to participate in neovascularization, tumor growth, and tumor invasiveness. While BMDCs are likely not the only contributing element to malignant transformation, targeting their recruitment to tumor sites has demonstrated efficacy in reducing tumor growth in animal models of other tumor types [22]. Furthermore, BMDCs are both practical and feasible as a therapeutic target and prognostic marker. Monitoring BMDC-related biomarkers in the peripheral blood for evidence of transformation may represent an improved prognostic tool over current methods, such as serial imaging, and may ultimately be less costly and easier to perform on a routine basis. Also, given that they are present in the peripheral blood, BMDCs would be particularly accessible as a potential therapeutic target, unprotected by the blood-brain barrier. Ultimately, improving our understanding of BMDCs in the context of neovascularization may have significant implications on improving clinical care of low-grade diffusely infiltrating gliomas and may provide the key target, that when leveraged properly, could prevent or delay the inevitable transformation to the high-grade, and ultimately fatal, phase of the disease.
